# Supervisor experience and cybersecurity response in aviation organizations using a two-wave exploratory design

**DOI:** 10.1371/journal.pone.0330942

**Published:** 2025-08-25

**Authors:** Filiz Mizrak, Kagan Cenk Mizrak, Umut Elbir

**Affiliations:** 1 Management Information Systems, Istanbul Atlas University, Istanbul, Turkiye; 2 Aviation Management, Istanbul Nisantasi University, Istanbul, Turkiye; 3 Occupational Health and Safety, Istanbul Bilgi University, Istanbul, Turkiye; Air Force Engineering University, CHINA

## Abstract

As cybersecurity threats continue to intensify across safety-critical industries, the aviation sector faces unique challenges due to its high digital dependency and human-centered operational environments. This study investigates how aviation supervisors in Türkiye perceive and respond to cybersecurity incidents involving employees, focusing on the psychological dimensions of leadership under pressure. Using a two-wave mixed-method design, data were collected from 300 supervisors through structured surveys and open-ended reflections on recent cybersecurity-related incidents. Thematic analysis identified common incident types such as phishing, password negligence, and unauthorized device use, along with eight distinct supervisory response strategies. Supervisors rated their actions on stressfulness and effectiveness, and K-means cluster analysis revealed three psychological response profiles. Multinomial logistic regression showed that psychological distress and time pressure were significant predictors of unfavorable experiences, while organizational cybersecurity support reduced the likelihood of high stress and low perceived effectiveness. These findings contribute to psychosocial risk and critical incident theory by highlighting cybersecurity incidents as emotionally significant events and underscore the need for supervisor training that integrates emotional resilience and cyber preparedness.

## 1. Introduction

The aviation sector, a cornerstone of global transportation, has become increasingly reliant on complex digital infrastructure. This reliance has, in turn, rendered the industry a prominent target for cyber threats. From airline booking systems and flight operations to air traffic control and passenger data, virtually every aspect of modern aviation is exposed to cyber risks. As Ukwandu et al. [1] highlight, the aviation industry’s growing digitization has made it highly vulnerable to diverse cyber-attack vectors, with threats ranging from ransomware to targeted intrusions. Similarly, Elmarady and Rahouma [2] stress that cybersecurity risks in civil aviation not only compromise information systems but may also lead to physical safety threats. Emerging attack types and hacker classifications, including hacktivists and cybercriminal groups, further escalate these challenges, making detection and prevention increasingly difficult [3].

Recent cases reinforce the urgency of addressing these risks. For instance, the 2020 cyberattack on EasyJet compromised the data of over 9 million customers, including financial details, leading to significant reputational damage and legal repercussions [4]. Similarly, the SITA Passenger Service System breach in 2021 affected multiple global airlines through a third-party vendor, exposing sensitive frequent-flyer data of millions of travelers. In 2022, SpiceJet, a major Indian carrier, suffered a ransomware incident that caused widespread flight delays [5], while Kuala Lumpur International Airport experienced operational paralysis due to a ransomware attack in 2023, forcing staff to revert to manual procedures [6]. Even Eurocontrol, Europe’s air traffic coordination body, was targeted by a sustained DDoS attack in 2023 that, while not affecting flight safety, disrupted communication systems and workflow efficiency [7]. These incidents illustrate how cyber threats can impact both data integrity and real-world operations, underlining the need for robust preventive and responsive strategies.

Despite global awareness efforts, cybersecurity readiness in aviation still varies widely, especially when it comes to human factors. Shah, Jhanjhi, and Brohi [8] argue that aviation cybersecurity cannot rely solely on technological countermeasures but must incorporate human-centric strategies. Recent studies emphasize the urgent need for a cohesive global defense framework in aviation cybersecurity [9], especially as passengers themselves grow more concerned about how airlines manage their cybersecurity responsibilities [10]. Mizrak and Akkartal [11] further underline the importance of prioritizing cybersecurity initiatives through strategic, data-driven methods that go beyond compliance-based approaches.

In this context, employee awareness and secure behavior emerge as critical components of aviation cybersecurity resilience. Human error remains a leading cause of cybersecurity incidents across industries, and aviation is no exception [12,13]. Studies show that employees who possess cybersecurity knowledge and demonstrate self-control are significantly more likely to engage in protective behaviors [14,15]. Moreover, customized cybersecurity training programs—especially those tailored to sector-specific needs—have proven effective in reducing security lapses attributed to human error [16]. Yet, as Alahmari, Renaud, and Omoronyia [17] argue, awareness alone is insufficient unless supported by mechanisms that encourage continuous knowledge sharing and reinforcement in the workplace.

At the frontline of this reinforcement are supervisors—key figures who shape employee behavior, enforce policy, and act during critical moments. The role of supervisors in strengthening cybersecurity culture is increasingly recognized. Their influence can guide employees toward better compliance and reduce risky behavior [18,19]. Supervisors not only transmit organizational expectations but also act as role models and informal educators, particularly when they respond directly to employee missteps. Mo and Chan [20] emphasize the importance of supervisory relationships in maintaining discipline and accountability in digital settings. According to Willie [21] a culture that encourages proactive supervisory engagement is fundamental to building a “security-first” workplace culture. Nevertheless, responding to cybersecurity lapses can be a complex and emotionally taxing responsibility. Supervisors may experience varying levels of stress, depending on organizational support, time pressure, and their own cybersecurity competency. Studies indicate that their responses are not always linear and may range from disciplinary action to coaching or even emotional support [22,23]. Understanding these varied responses requires more than just outcome-based evaluation—it necessitates a situational, context-sensitive lens.

To that end, this study adopts a Critical Events Approach (CEA), inspired by Jimmieson and Bergin’s [24] framework, which explores how supervisors perceive and respond to significant work events that require psychological adaptation. CEA focuses on the intensity, novelty, and disruptiveness of incidents and the responses they provoke. While previously applied in contexts such as psychosocial risk and employee well-being, the approach offers a novel lens for understanding the human dynamics of cybersecurity event management in aviation. The value of CEA is echoed in recent applications in other high-risk sectors such as railway security [25], reinforcing its relevance to safety-critical environments like aviation. Based on this conceptual foundation, the study addresses the following research questions:

- What actions do aviation supervisors take in response to cybersecurity lapses?
- How do supervisors perceive these actions in terms of stress and effectiveness?
- What factors shape supervisors’ psychological responses during cybersecurity-related critical events

By exploring these questions, the study aims to uncover actionable insights that contribute to aviation cybersecurity resilience through improved supervisory practice and human-centered policy development. In doing so, the study makes the following key contributions to the literature:

- It is among the first to apply the Critical Events Approach (CEA) to cybersecurity-related incidents in the aviation sector, emphasizing the psychological framing of supervisory interventions.
- It integrates psychosocial risk constructs—such as emotional strain, psychological distress, and time pressure—with organizational predictors of supervisor behavior, offering a multidisciplinary perspective.
- It employs a two-wave mixed-method design that temporally separates pre-incident conditions from incident-specific reflections, enhancing the ecological validity of findings.
- It proposes and validates a typology of eight supervisory response strategies, filling a gap in the taxonomy of leadership behaviors in cybersecurity event management.
- It introduces a novel 2 × 2 matrix mapping supervisor actions by perceived stress and effectiveness and complements this with K-means cluster profiling, offering a new evaluative lens for cyber leadership.
- It provides practical implications for aviation-specific cyber training and emotional resilience development, helping organizations enhance supervisor preparedness beyond traditional compliance models.

These contributions collectively offer a fresh, empirically grounded perspective on the intersection of cybersecurity, leadership, and psychological resilience in one of the world’s most safety-critical industries.

## 2. Literature review

### 2.1. Major cybersecurity cases in aviation (2020–2025)

The aviation industry has faced a surge of cybersecurity threats in recent years, underlining its growing vulnerability to cyberattacks that threaten operational continuity, data integrity, and passenger trust. From data breaches and ransomware to denial-of-service and supply chain attacks, these incidents have prompted a re-evaluation of digital resilience within aviation ecosystems. One of the earliest and most significant incidents in this period was the EasyJet data breach in 2020, where a sophisticated cyberattack compromised the personal data of over nine million customers, including 2,208 cases of credit card theft [4]. The delayed disclosure and scale of the breach led to substantial reputational damage and regulatory scrutiny. This event emphasized the necessity for stronger data protection protocols and more timely incident reporting mechanisms. Another major event was the SITA Passenger Service System breach in 2021, which exemplified the risks posed by third-party vendors. The breach affected millions of frequent flyer accounts across several global airlines, including Lufthansa, Air New Zealand, and Singapore Airlines [4]. The incident highlighted the interconnectedness of airline IT infrastructures and the critical importance of supply chain cybersecurity management. The ransomware attack on SpiceJet in 2022 disrupted flights across India, demonstrating how operational technologies (OT) are now frequent targets. Despite the attack being contained within hours, it caused significant passenger delays and highlighted the value of rapid incident response and robust backup systems [5].

Airport infrastructure has also been a prominent target. For example, San Francisco International Airport (SFO)experienced a breach in 2020 where attackers compromised employee web portals to steal credentials [26]. Similarly, Kuala Lumpur International Airport (KLIA) faced a ransomware attack in 2023 that crippled operational systems and forced the airport to revert to manual processes, raising questions about the preparedness of aviation hubs against digital extortion [6]. The Kenya Airports Authority breach in 2023 revealed another dimension: the exposure of internal blueprints, staff data, and critical infrastructure details, posing long-term security risks beyond immediate operational disruption [27]. These incidents underscore the need for holistic cybersecurity strategies encompassing IT, OT, and personnel systems.

Air traffic control organizations have not been immune. In 2023, Eurocontrol, Europe’s ATC coordination body, suffered a prolonged DDoS attack linked to geopolitical tensions. Though flight safety was maintained, operational workflows and communication channels were severely hampered, requiring fallback to analog methods such as fax [7]. This case revealed that even without penetrating flight control systems, cyberattacks can degrade essential coordination infrastructure. Moreover, hacktivist campaigns like those from Killnet targeted over 40 U.S. airport websites in 2022, rendering them inaccessible to travelers without affecting core aviation systems [27]. These attacks, often dismissed as mere annoyances, nevertheless expose the fragility of public-facing systems and the need for DDoS mitigation. Additionally, the Japan Airlines DDoS attack in 2024 caused delays in over 20 domestic flights during peak travel time, reaffirming that non-invasive cyberattacks can still impact operational continuity and passenger service [28].

Collectively, these cases reveal evolving patterns in aviation cybersecurity: a rise in politically motivated attacks, increasingly sophisticated ransomware campaigns, and the targeting of weak points in both digital and human systems. The incidents between 2020 and 2025 serve as both a cautionary tale and a roadmap for fortifying cyber resilience in aviation.

### 2.2. Cybersecurity as a psychosocial challenge

Cybersecurity in the contemporary workplace, particularly in high-stakes industries such as aviation, can no longer be understood purely as a technical concern. It increasingly embodies complex psychosocial dynamics that influence both employee behavior and organizational resilience. The aviation sector’s digital transformation—marked by the proliferation of interconnected systems, automated decision-making tools, and reliance on real-time data—has ushered in an era where supervisors are not only operational leaders but also key agents of cybersecurity culture. This shift introduces significant cognitive and emotional demands, particularly in moments where supervisors are required to intervene in cybersecurity-related incidents involving their teams. These moments, while often technical in nature, are experienced as critical psychological events, requiring immediate assessment, action, and often interpersonal negotiation.

Recent psychological research offers strong evidence that cyberspace presents numerous cognitive and emotional stressors that go beyond system-level challenges. Khawrin and Nderego [29], in their systematic review of meta-analyses, identify a range of psychological burdens associated with cyberspace—such as decision fatigue, cognitive overload, anxiety, and a diminished sense of control. These burdens are especially salient in work contexts where individuals are expected to maintain constant vigilance, adhere to evolving security protocols, and remain responsive to unpredictable digital threats. In aviation environments, where every decision may carry implications for safety and operational integrity, such psychological demands are magnified. Supervisors, positioned between frontline employees and upper management, often face the brunt of these demands without adequate psychological or procedural support. Furthermore, recent technical studies have emphasized the increasing complexity of cyber threats targeting aviation communication, navigation, and surveillance systems—commonly referred to as CNS infrastructures [30]. These foundational systems are subject to sophisticated adversarial attacks, heightening the pressure on supervisors during anomaly detection and incident response.

Cybersecurity lapses, particularly those involving human error, have been shown to trigger stress responses similar to psychosocial risk events, especially for those responsible for incident containment and mitigation. The work of McAlaney, Taylor, and Faily [31] emphasizes that cybersecurity behaviors are shaped by social context, perceived risk, and emotional states, making digital threats as much psychological as technical. This perspective is echoed by Vila et al. [32], who argue that organizational culture, peer behavior, and stress levels significantly influence compliance with cybersecurity protocols. Supervisors navigating such incidents are not merely enforcing policy; they are managing conflict, emotional fallout, reputational risk, and their own internal responses—all of which can impact the quality and effectiveness of their interventions. When a team member, for example, inadvertently exposes the organization to phishing or data breaches, it is the supervisor who must simultaneously address the incident, correct the behavior, maintain team morale, and report upward—often in real time. These emotionally charged scenarios are further complicated by novel cyberattack modalities such as adversarial false data injection [33,34], which target the integrity of AI-driven monitoring systems and reduce clarity in supervisory decision-making under time constraints.

This human dimension of cybersecurity is particularly pronounced in critical incident scenarios. Jimmieson and Bergin [24] describe critical work events as those that are novel, disruptive, and demanding—qualities that clearly align with cybersecurity failures in aviation. Such events disrupt routine operations and demand rapid supervisory judgment, which can either reinforce or undermine organizational resilience. The parallels with psychosocial risk events are strong. Both types of events are emotionally charged, demand situational leadership, and often require navigating ambiguity, blame, and uncertainty. Supervisors may experience internal conflict over how to balance organizational expectations with support for the employee, especially when there is a lack of clear guidance or sufficient cybersecurity knowledge. This ambiguity has been linked to emotional exhaustion, reduced confidence, and inconsistent decision-making [35]. Advanced attacks—such as those targeting flight trajectory prediction with dynamic perturbations **[**36**]**—further blur the boundary between technical disruption and human stress, demanding high adaptability from supervisors.

Furthermore, digital threat landscapes are becoming more socially embedded and behaviorally complex. Patel and Doshi [37] introduce the concept of the Internet of Behavior (IoB), which illustrates how personal behaviors, online habits, and emotional reactions contribute to cyber risk exposure. Their work suggests that secure behavior is not only a function of policy but of psychological literacy and emotional regulation. Supervisors who are attuned to these behavioral cues may be more effective at preventing incidents, but they are also more susceptible to emotional burden when incidents do occur. In addition, the increasing use of psychological profiling and psycholinguistic analysis in cybersecurity—such as that discussed by Tshimula et al. [38]—demonstrates that modern cyber risk management strategies are shifting toward more individual-centric approaches that require empathy, real-time judgment, and behavioral insight from leaders. Morhunova [39], in her study on data protection in remote psychological services, underscores the psychological fragility of digital interactions, drawing attention to the emotional consequences of data breaches and loss of digital trust. These consequences are not confined to therapy contexts; in aviation, breaches in cyber hygiene can similarly affect professional trust, team dynamics, and employee-supervisor relationships. When a cybersecurity lapse occurs, it may not only compromise operational systems but also erode the psychological safety of the workplace. For supervisors, this erosion can create moral stress, especially when they are expected to discipline or report employees they are also meant to mentor and support. Additionally, aviation networks increasingly rely on interconnected service functions, which—if disrupted by targeted attacks—can generate cascading failures across operational systems [40], adding systemic strain to supervisory decision-making.

Given these dynamics, cybersecurity lapses in aviation should be viewed not merely as technical errors, but as psychosocial disruptions that can affect organizational performance, employee well-being, and supervisory integrity. These events demand a deeper understanding of how supervisors experience, interpret, and respond to such situations. The Critical Events Approach (CEA) offers a valuable framework for capturing this complexity. As applied in high-reliability sectors [24,25], CEA enables researchers to focus on the subjective experiences of those directly involved in managing disruptive events. This approach allows for a more nuanced understanding of the stressfulness and perceived effectiveness of supervisor actions, and how these responses are shaped by pre-existing factors such as time pressure, organizational support, and personal distress.

In summary, cybersecurity breaches, especially those arising from employee behavior, should be recognized as critical psychosocial events within aviation organizations. They represent moments of heightened vulnerability—not just for IT infrastructure but for interpersonal relationships, trust in leadership, and organizational culture. Supervisors, often under-supported and undertrained in the psychological aspects of digital risk management, are central to how these events are handled and how resilient the organization ultimately becomes. To address this gap, the current study applies the Critical Events Approach to explore how supervisors in aviation experience, respond to, and evaluate cybersecurity-related incidents involving their teams, with the aim of contributing to both theoretical insight and practical guidance in cybersecurity leadership.

### 2.3. Supervisory actions in cybersecurity context

The supervisory role in cybersecurity has evolved significantly, extending beyond technical oversight to include proactive management of human behavior, real-time decision-making during incidents, and post-event psychological support. In aviation organizations—where operational continuity and safety are paramount—supervisors serve as the first line of response when employees engage in risky digital behavior or trigger cybersecurity incidents. This dual role requires both technical literacy and psychosocial competence, making it imperative to understand not only what actions supervisors take, but also the cognitive, behavioral, and emotional frameworks that shape their decision-making.

Historically, cybersecurity frameworks such as Supervisory Control and Data Acquisition (SCADA) systems were developed to detect anomalies and unauthorized access in industrial infrastructure. Cruz et al. [41] proposed a cybersecurity detection architecture tailored for SCADA environments that emphasizes real-time supervisory intervention, signaling the growing need for human-in-the-loop oversight even in highly automated systems. While primarily technical, the model illustrates a critical theme: supervisory roles in cybersecurity must be responsive, adaptive, and informed by contextual cues. Rasouli, Miehling, and Teneketzis [42] advance this argument by integrating dynamic game theory into supervisory control, enabling supervisors to predict adversarial behavior and adjust strategies accordingly. Although developed in control system environments, such frameworks are increasingly relevant in high-risk aviation settings, where supervisors must make fast, high-stakes decisions based on partial information during cyber incidents.

In more human-centered contexts, research by Lau et al. [43] and Hadjicostis et al. [44] has shown that effective supervisory cybersecurity responses require situational awareness, accurate risk estimation, and resilience under stress. Lau et al. [43] underscore the need for supervisors to understand human-computer interaction in their control processes, particularly during recovery from attacks. Van der Kleij et al. [45] take this further by proposing decision support tools for cybersecurity incident managers, suggesting that timely and psychologically attuned decision-making is as important as technical countermeasures. These findings align with the aviation sector, where supervisors often operate under tight time constraints, with limited cybersecurity training, and in environments where errors can cascade into safety threats.

While these technical models emphasize detection and control, the human and organizational dimensions of supervisory action are equally important—particularly in responding to employee-caused cyber incidents. This study proposes adapting the Psychosocial Risk Management (PSRM) framework to cybersecurity. Traditionally used to manage stress, interpersonal conflict, and safety culture, PSRM provides a structure for assessing psychological demands, support systems, and individual coping mechanisms within work environments [46]. In the aviation context, applying PSRM means recognizing that a supervisor’s reaction to a cybersecurity lapse is not purely procedural, but shaped by emotional load, perceived organizational support, role clarity, and personal resilience.

Cybersecurity literature has increasingly recognized these dynamics. Nygard et al. [47] identify “organizational factors” such as communication, leadership consistency, and perceived fairness as critical to effective cybersecurity risk management. Similarly, Jose, LaPort, and Trippe [48] argue that cybersecurity teams must possess not only technical skills but psychosocial attributes like adaptability, empathy, and emotional regulation—qualities especially vital for supervisory roles. Greitzer and Frincke [49] advocate combining behavioral data with audit logs to anticipate insider threats, emphasizing the psychosocial footprint of risky digital behavior. This suggests that supervisors, who are closest to day-to-day operations and interpersonal dynamics, are in a unique position to both detect and mitigate these threats—if properly supported.

Supervisory responses to cybersecurity lapses can take many forms, and literature points toward the need for a taxonomy of response strategies. Based on findings from both technical and psychosocial domains, these strategies can be classified along several dimensions:

- Corrective vs. Supportive – Some supervisors adopt a disciplinary stance, issuing formal warnings or escalating incidents to HR or IT. Others choose coaching, follow-up training, or informal mentoring as their primary response [19,21].
- Reactive vs. Proactive – Supervisors may act only after a breach occurs, or they may anticipate risks by regularly reinforcing security protocols, conducting drills, and sharing updates [18].
- Directive vs. Participative – While some supervisors enforce rules with little dialogue, others engage employees in discussions, emphasizing shared responsibility and reflective learning [20].
- Stress-Inducing vs. Stress-Buffering – The way in which a supervisor communicates during and after an incident can either escalate emotional strain or provide psychological safety, which in turn affects future compliance and team cohesion [24,31].

These response types are not mutually exclusive and are often shaped by contextual variables such as organizational support, supervisor training, time pressure, and psychological distress—factors also measured in the current study. Importantly, supervisors may not perceive their own responses uniformly. A decision to issue a warning might be seen as necessary but emotionally taxing, while a coaching conversation may be experienced as more effective and less stressful, even if it lacks immediate enforcement power.

The Critical Events Approach, used in this study, provides a valuable structure for capturing these subjective experiences. By collecting supervisor narratives and measuring their perceptions of stressfulness and effectiveness, the approach allows for mapping these responses into clusters—such as favorable (low stress, high effectiveness), challenge (high stress, high effectiveness), and unfavorable (high stress, low effectiveness). This typology, informed by both technical and psychosocial frameworks, will support the development of actionable insights into how aviation supervisors can better manage cybersecurity lapses among employees while preserving both organizational security and psychological well-being.

### 2.4. Supervisors’ psychological responses

The psychological dimension of supervisory work has taken on renewed significance in the digital age, especially in high-stakes sectors like aviation, where supervisory actions have both operational and safety implications. In cybersecurity incidents triggered by employee behavior—such as phishing, data mishandling, or device misuse—supervisors are often thrust into rapid decision-making roles. These situations carry a heavy emotional and cognitive load. They demand clarity, composure, and leadership in moments marked by ambiguity, pressure, and potential reputational risk. In such contexts, the subjective experiences of supervisors—how they evaluate the stressfulness of a situation and the effectiveness of their responses—become vital indicators of both their psychological well-being and their leadership impact.

Drawing on the Critical Events Approach (CEA), Jimmieson and Bergin [24] emphasize that supervisors’ responses to employee-related work stressors are not uniform; rather, they reflect varied emotional and cognitive appraisals of the same event. Their study, which forms the methodological foundation of the current research, found that while some supervisors perceived their interventions as effective and minimally stressful, a significant portion—nearly 48%—classified their experiences as both highly stressful and highly effective, identifying them as challenge experiences. These situations are psychologically demanding but also meaningful, reinforcing the supervisor’s sense of role importance and agency. Supervisors who fell into this category often reported that although the incident caused temporary emotional strain, it was ultimately affirming of their leadership capacity. This dual experience—what Rothman et al. [50] called “emotional ambivalence”—can be productive, fostering growth and engagement, but only when organizations provide space for reflection and psychological processing.

The emotional exhaustion associated with managing these incidents is often compounded by organizational rigidity. Charoensukmongkol and Phungsoonthorn [51], in their study of university employees during the COVID-19 crisis, found that while supervisor support reduced emotional exhaustion, this benefit was undermined in organizations characterized by intransigence—rigid hierarchies, resistance to change, and poor communication flows. In aviation cybersecurity, this is highly relevant. Supervisors often face bureaucratic constraints when responding to cyber incidents, such as slow IT escalation pathways or unclear protocols for handling minor breaches. These factors can increase the emotional cost of supervision, especially when a supervisor’s intentions conflict with procedural limitations. The tension between policy compliance and immediate decision-making responsibility is a core source of psychological stress in cybersecurity event management.

Adding a motivational perspective, Dai et al. [52] found that supervisors with strong psychological ownership—the belief that their work matters and that they are stewards of organizational success—experience greater engagement and less burnout, even under pressure. They also highlighted the importance of supervisors’ organizational embodiment, or how closely supervisors feel aligned with the values and identity of the organization. In cybersecurity settings, when a supervisor feels genuinely responsible for the integrity of systems and the learning of their employees, the stress of managing a digital incident may be offset by a sense of purpose and impact. However, when organizational culture fails to recognize or reward such psychological investment, the same situations can lead to frustration and disengagement. Supervisors who view themselves as passive executors of external policy rather than proactive agents of cybersecurity culture are more vulnerable to burnout and perceived ineffectiveness, particularly when handling incidents involving recurring human error.

Moreover, the source of stress may not always be the incident itself, but the expectations placed upon the supervisor—both from the organization and from subordinates. McLarty et al. [53] introduced the concept of “supervisor-induced hindrance stressors,” whereby leaders who lack clarity, consistency, or emotional intelligence contribute to job neglect and performance decline—not only in their subordinates but also in themselves. Supervisors who internalize failure, or who lack sufficient relational capital with their teams, are more likely to experience these events as overwhelming and unrewarding. In cybersecurity, where a small error can trigger significant technical consequences, the fear of being blamed or judged may further exacerbate psychological pressure.

The complexity of supervisory roles in cybersecurity is further highlighted by studies on humanization and perceived authority. Yam et al. [54] found that individuals reacted more emotionally—often more spitefully—toward robotic supervisors that mimicked human behavior, compared to those that were clearly non-human. This suggests that emotional expectations are heightened when authority figures are perceived as relatable. By extension, in aviation, human supervisors carry a relational burden; their decisions are evaluated not only for correctness but for fairness, empathy, and consistency. This increases the likelihood of supervisors experiencing moral stress, especially when required to discipline employees they have mentored or supported. Furthermore, the perception of effectiveness is not always aligned with emotional comfort. Supervisors may execute a policy correctly and still feel uncertain about the interpersonal impact of their decision. For example, coaching an employee after a phishing lapse may be less emotionally taxing but may not satisfy formal compliance measures, whereas issuing a warning may achieve procedural clarity but leave the supervisor emotionally conflicted. Jimmieson and Bergin [24] found that such subjective evaluations of effectiveness are highly contextual and influenced by supervisors’ perceived organizational support, personal values, and psychological state at the time of the incident. Supervisors with lower psychological distress, measured using scales like the Kessler K10, were more likely to perceive their actions as effective and less stressful, suggesting that internal resilience plays a protective role in high-stakes supervisory decision-making.

Altogether, the psychological responses of supervisors to cybersecurity events are shaped by an intricate mix of emotional regulation, cognitive appraisal, organizational environment, and relational context. These responses influence not only the supervisor’s well-being but also the consistency and quality of cybersecurity enforcement across the organization. As such, aviation institutions must go beyond technical training and invest in psychosocial support structures for supervisors. These could include reflective supervision models, leadership development focused on emotional intelligence, and mechanisms for debriefing after critical cybersecurity events. Understanding supervisory psychological responses as part of cybersecurity resilience strategy is essential for building a robust digital safety culture in aviation.

### 2.5. Pre-existing feature variables

In assessing how aviation supervisors respond to cybersecurity-related critical events, it is essential to consider the pre-existing psychological and organizational conditions that shape their interpretations, decisions, and perceived effectiveness. These antecedent variables do not occur in isolation but interact dynamically with the supervisor’s experience of the event. Prior research in occupational psychology and cybersecurity management has identified several key variables that significantly influence how individuals interpret stress, manage responsibility, and evaluate their own leadership actions. In the context of this study, four variables—organizational cybersecurity support, supervisor cybersecurity knowledge, time pressure, and psychological distress—are included to examine their predictive relationship with supervisors’ perceptions of stressfulness and effectiveness during cybersecurity incidents.

*Organizational Cybersecurity Support-*Organizational cybersecurity support reflects the perceived presence of resources, guidance, leadership commitment, and a security-conscious culture within the organization. This construct is informed by broader research into cybersecurity culture and organizational safety practices. According to the Infosec Institute’s *Cybersecurity Culture Survey* [55], organizations that regularly provide communication about threats, make security contacts visible, offer training, and empower supervisory personnel to take ownership of cybersecurity foster a more engaged and vigilant workforce. In such environments, supervisors are more likely to feel confident when handling cybersecurity incidents and are less prone to stress related to ambiguity or isolation. In the aviation sector, where human error remains one of the most persistent vectors for cyber threats, the absence of visible cybersecurity leadership or clear procedural support can significantly undermine supervisors’ ability to respond effectively. Mizrak and Akkartal [11] emphasize the importance of prioritizing cybersecurity initiatives through structured models, yet without frontline implementation support, these initiatives may lack practical impact. As demonstrated in the Jimmieson and Bergin’s study [24], perceived organizational PSRM (psychosocial risk management) was associated with lower stress and higher response confidence among supervisors, suggesting that perceived institutional commitment is critical to supervisory resilience.

*Supervisor Cybersecurity Knowledge-*Another crucial precondition is the supervisor’s own cybersecurity knowledge—encompassing technical understanding, awareness of policies, and ability to communicate secure practices to others. This construct is especially relevant in light of findings by Santhosh and Thiyagu [56], who developed a Cybersecurity Competency Scale to evaluate how knowledge of secure behaviors, risk assessment, and digital hygiene contributes to professional confidence. Although their study targeted prospective teachers, its applicability is broad, particularly in supervisory contexts where individuals are responsible for enforcing organizational rules and correcting unsafe behavior. Supervisors with stronger cybersecurity knowledge are likely to feel more empowered during incidents, better able to interpret technical risks, and less prone to internalizing blame or uncertainty. In the context of aviation, this is especially important given the hybrid nature of cybersecurity challenges—where both physical and digital domains intersect. Supervisors must not only understand procedures but also recognize phishing attempts, respond to insider threats, and explain password or device protocols to their teams. The absence of these competencies may exacerbate stress, especially during incidents involving operational systems or data integrity.

*Time Pressure (HSE Management Standards Indicator Tool)-* Time pressure is another widely recognized antecedent to stress in organizational environments and is measured here using the Health and Safety Executive (HSE) Management Standards Indicator Tool [57]. The tool captures perceptions of workload demands, conflicting deadlines, and the adequacy of time to complete tasks—all factors that are especially relevant in aviation, where operations are fast-paced, time-bound, and safety-critical. Supervisors experiencing chronic time pressure may find it difficult to allocate attention to cybersecurity training, policy enforcement, or incident management. Moreover, under time-constrained conditions, supervisors may be more likely to adopt reactive rather than proactive strategies, opting for expedient decisions that may not be psychologically or procedurally optimal. Prior studies in stress management (e.g., Saik et al., [46]) indicate that time pressure not only impacts cognitive performance but also reduces leaders’ emotional bandwidth to offer support or communicate effectively during a crisis. In cybersecurity contexts, this can result in lower response quality and higher stress, particularly if supervisors perceive that cybersecurity tasks interfere with operational efficiency.

Psychological Distress (Kessler K10 Scale)- Lastly, this study includes psychological distress as a predictor of supervisory response outcomes, measured using the Kessler Psychological Distress Scale (K10) developed by Kessler and Mroczek [58]. The K10 is a widely used screening tool for assessing non-specific psychological distress, including symptoms such as fatigue, hopelessness, nervousness, and restlessness. In high-pressure professional environments, elevated levels of psychological distress can impair judgment, reduce emotional regulation, and distort supervisors’ appraisal of their actions and environment. Jimmieson and Bergin [24] found that supervisors with higher baseline distress were more likely to perceive their responses to employee stressors as both less effective and more stressful, regardless of the objective success of their intervention. In aviation cybersecurity, where supervisors may already be under considerable strain due to regulatory pressure, personnel shortages, or technical challenges, distress becomes a significant moderator of perceived leadership efficacy. Including psychological distress as a feature variable in this study acknowledges that the internal emotional state of the supervisor is a crucial determinant of how critical events are interpreted and acted upon.

In summary, the inclusion of these four pre-existing variables—organizational cybersecurity support, supervisor cybersecurity knowledge, time pressure, and psychological distress—offers a comprehensive view of the supervisory ecosystem in aviation cybersecurity management. These variables are not only theoretically grounded in occupational and cyber-psychological research but also practically relevant for designing targeted interventions aimed at improving both incident response quality and supervisor well-being.

## 3. Methodology

### 3.1. Research design

This study employs a mixed-method, two-wave sequential research design to investigate how aviation supervisors respond to cybersecurity-related critical events involving employees. The research is grounded in the Critical Events Approach (CEA), enabling the exploration of both subjective supervisory experiences and the broader organizational and psychological variables that shape their responses. The study received ethical approval from the Ethics Committee of Beykoz University on 07 June 2024 (Approval No: E-45152895-299-2400008120) and was conducted in accordance with institutional ethical guidelines. All stages of the research ensured participant confidentiality and data privacy. Participant recruitment was carried out over a three-month period following ethics approval, beginning on 10 June 2024 and concluding on 10 September 2024.

The first wave (T1) comprised a structured quantitative survey that captured supervisors’ perceptions of organizational cybersecurity support, self-assessed cybersecurity knowledge, time pressure, and psychological distress. These variables were measured using validated instruments, including adaptations of the Infosec Cybersecurity Culture Survey, Santhosh and Thiyagu’s Cybersecurity Competency Scale, the Health and Safety Executive (HSE) Time Pressure Indicator Tool, and the Kessler K10 Psychological Distress Scale. This phase established a foundational understanding of the supervisory context in which cybersecurity incidents occur, serving as a baseline for interpreting the findings of the second wave.

Conducted approximately four weeks after T1, the second wave (T2) integrated both qualitative and quantitative elements. Supervisors were asked to recall and describe a recent, meaningful cybersecurity incident involving an employee—conceptualized as a “critical event”—and to outline the actions they took in response. This qualitative component offered deep insights into real-world decision-making, leadership behavior, and emotional tone. Alongside the open-ended narratives, supervisors rated their responses on two 10-point Likert scales: one assessing the perceived stressfulness of their response, and the other its perceived effectiveness.

**Fig 1:**
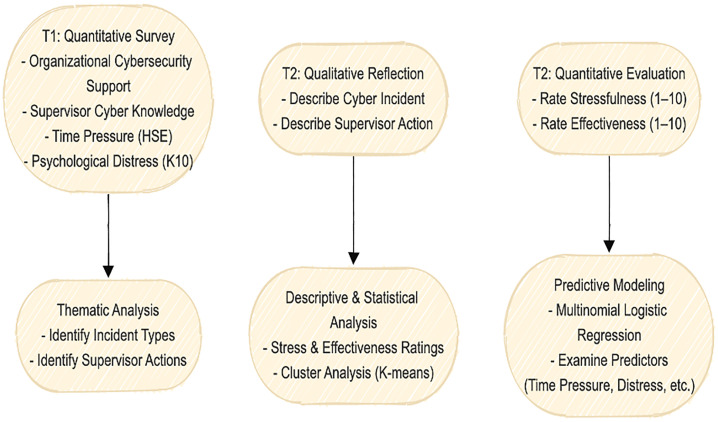
Workflow of the two-wave mixed-method study design.

The sequential structure of the design allows for the temporal separation of pre-existing psychological and organizational factors (T1) from incident-specific reflections (T2). This separation helps minimize bias and provides a clearer understanding of how earlier conditions may influence later supervisory responses. By combining quantitative predictors with rich qualitative data, the study offers a holistic perspective on supervisory behavior—balancing generalizability with contextual depth. This design also facilitates advanced analyses such as thematic coding and cluster identification, enabling the categorization of supervisory responses into types such as “favorable,” “challenge,” or “unfavorable,” as outlined in previous research (Jimmieson & Bergin, [24]. To illustrate the methodological progression and data collection timeline, Fig 1 presents a visual overview of the mixed-method, two-wave sequential design employed in this study.

While the overall design is sequential, the findings section begins with thematic analysis of the qualitative narratives collected in T2. This ordering was chosen intentionally, as supervisors’ descriptions of employee incidents and their own actions provide the contextual foundation for interpreting later ratings of stressfulness and effectiveness. Presenting the qualitative categories first also aligns with the Critical Events Approach, which emphasizes understanding the nature of the event and the response before quantifying psychological appraisal. This structure allows the reader to understand what happened, how supervisors reacted, and then how they experienced those reactions—ensuring coherence between methodology, theory, and reporting. This methodology is particularly suitable for high-risk, fast-paced environments like aviation, where cybersecurity lapses can be sudden, emotionally charged, and operationally significant, and where supervisors are often the first line of both prevention and response.

### 3.2. Participants and sampling

Participants were recruited through purposive sampling strategies, including outreach via aviation-focused HR networks, online research platforms, and direct partnerships with industry contacts in Türkiye. Eligible participants were required to hold a supervisory or managerial position within the aviation industry, spanning roles in airlines, airports, and ground operations. Emphasis was placed on individuals actively involved in team oversight, employee performance management, and the implementation or enforcement of cybersecurity policies. At Time 1 (T1), a total of 345 responses were collected through an online survey administered in Turkish. Among these, 300 participants (87%) confirmed that they held a current supervisory role, while the remaining 45 were excluded from the Time 2 (T2) phase. Within the T1 sample, 229 supervisors (76.3%) reported having received formal cybersecurity awareness training, while 116 supervisors (38.7%) indicated that they had not received such training. The Time 2 (T2) stage involved a critical event recall and reflection. Supervisors were asked to describe a recent cybersecurity-related incident involving an employee and report their actions, as well as rate their perceived stressfulness and effectiveness of the response. After screening for missing or uninterpretable entries, 300 matched supervisor responses were retained for full analysis. Descriptive statistics from the T1 dataset are summarized in Table 1.

**Table 1:** Supervisor demographics summary.

Characteristic	Value
Total Participants	345
Supervisors (Yes)	300
Supervisors (No)	45
Average Years of Experience	16.28
Minimum Experience (Years)	1
Maximum Experience (Years)	30
Cyber Training Received (Yes)	229
Cyber Training Received (No)	116

The variety of roles and professional backgrounds enhances the validity of the findings and supports both the quantitative (T1) and qualitative (T2) dimensions of the study.

### 3.3. Instruments and measures

At Time 1 (T1), supervisors completed a structured survey comprising multiple validated and adapted instruments designed to assess their perceptions of organizational cybersecurity preparedness, their own cybersecurity knowledge and confidence, work-related time pressure, psychological distress, and general cybersecurity behavior. To evaluate perceptions of organizational cybersecurity support, seven items were adapted from prior IT governance and cybersecurity culture surveys, including sources such as the Infosec Institute’s Cybersecurity Culture Survey. These items measured the extent to which supervisors believed their organizations provided clear guidance, visible resources, and ongoing support for cybersecurity practices. Participants rated statements such as “My organization provides clear protocols for reporting cyber incidents” on a 7-point Likert scale from 1 (strongly disagree) to 7 (strongly agree). Exploratory factor analysis (EFA) supported a unidimensional scale (eigenvalue = 4.81), explaining 68.7% of the variance, with high internal consistency (α = 0.91).

To assess supervisor cybersecurity knowledge and confidence, six items were adapted from Santhosh and Thiyagu’s [56] Cybersecurity Competency Scale. Items reflected technical knowledge, risk identification skills, and confidence in applying and communicating cybersecurity protocols. Participants responded to items such as “I feel confident addressing employees about secure digital behavior” using a 7-point agreement scale. An EFA supported the internal consistency of the scale (α = 0.88), with a single factor accounting for 65.4% of the variance. Time pressure was measured using three items from the demand subscale of the UK Health and Safety Executive (HSE) Management Standards Indicator Tool (2004), which captures perceptions of workload and unreasonable time constraints. Participants rated how frequently they experienced pressures such as “I have unrealistic time pressures” on a scale from 1 (never) to 7 (always). This scale demonstrated high reliability with an alpha coefficient of α = 0.86.

Supervisors’ psychological distress was assessed using the Kessler Psychological Distress Scale (K10), a 10-item measure designed to capture general symptoms of anxiety, hopelessness, and fatigue experienced in the past four weeks. Respondents rated items like “How often did you feel so restless you could not sit still?” on a 5-point scale ranging from 1 (none of the time) to 5 (all of the time), with higher scores indicating greater distress. The scale showed strong internal consistency in this sample (α = 0.89). In addition to these workplace and psychological variables, supervisors’ cybersecurity behavior and awareness were measured using a hybrid of the Security Behavior Intentions Scale (SeBIS) and selected items from the Cybersecurity Awareness Questionnaire (CAQ). These items examined proactive behavior, password security, awareness of potential threats, and keeping systems up to date. Participants responded to items such as “I make an effort to stay informed about new security threats” on a 5-point scale. The combined behavioral scale showed multidimensionality across four subscales—password practices, device security, update vigilance, and proactive awareness—with all subscales demonstrating internal consistency coefficients above α = 0.80.

At Time 2 (T2), which took place four weeks after the initial survey, participants were asked to recall a real cybersecurity-related event involving an employee. Supervisors provided a narrative account of the event and the action they took in response. In addition to the open-ended responses, participants rated the event on two 10-point Likert scales: one assessing how stressful they found their experience of responding to the incident (1 = not at all stressful, 10 = extremely stressful), and the other assessing how effective they believed their response was (1 = not at all effective, 10 = extremely effective). This design enabled the study to link supervisors’ pre-existing psychological and organizational conditions (T1) with their real-world reflections on cybersecurity incidents (T2), supporting both predictive and thematic analyses in subsequent stages.

## 4. Results

### 4.1. Data analysis approach

To address the first aim of the study—namely, to identify the types and frequencies of cybersecurity-related incidents involving employees, along with the range of supervisory actions taken in response—qualitative data collected from 300 supervisors at Time 2 were subjected to thematic analysis [59]. This analysis focused on two sets of open-ended responses: (1) the description of the cybersecurity incident involving an employee, and (2) the supervisor’s corresponding response or intervention. Thematic analysis followed the methodological framework established by Braun and Clarke [60], which involves iterative reading, coding for emergent patterns, and developing discrete categories that reflect shared meanings across narratives.

The analysis was conducted by the first author and second author with prior experience in cybersecurity behavior and digital safety culture. Both raters independently coded the responses and then compared interpretations. Coding differences were discussed and reconciled through consensus, after which the codes were collated into higher-order themes such as types of cybersecurity breaches (e.g., phishing, password misuse, unauthorized device use) and forms of supervisor action (e.g., coaching, reprimanding, escalation to IT, informal warning).

Prior to thematic analysis, each open-ended response was evaluated for response effort and elaboration quality. This assessment was performed by the same two coders, who rated the narrative responses on a 3-point scale: 1 (minimal effort), 2 (moderate detail), and 3 (highly elaborated and reflective). The inter-rater agreement was strong, with a Cohen’s kappa of 0.93 (p < .001). After discussion of discrepancies, 47 responses were rated as low effort, 128 as moderate, and 125 as high. All responses were retained for thematic coding, though lower-effort responses were interpreted with additional caution during qualitative interpretation.

To examine the relationships between types of cybersecurity incidents and supervisory responses, a cross-tabulation and chi-square analysis was conducted to determine whether certain forms of supervisor action were more likely to follow specific incident types. Furthermore, to explore whether perceived stressfulness and effectiveness varied as a function of the type of supervisory response, an analysis of variance (ANOVA) was performed. These data were also mapped onto a 2 × 2 stress-effectiveness matrix, classifying actions into categories such as *favorable* (low stress, high effectiveness), *challenge* (high stress, high effectiveness), *unfavorable* (high stress, low effectiveness), and *neutral* (low stress, low effectiveness).

To address the second aim—understanding how pre-existing feature variables (measured at Time 1) predicted perceived stressfulness and effectiveness—bivariate correlations were first calculated. Variables included organizational cybersecurity support, supervisor cyber knowledge, time pressure (HSE), and psychological distress (K10). To examine how combinations of these predictors influenced supervisors’ likelihood of falling into one of the four stress-effectiveness categories, a two-step cluster analysis was conducted, followed by a series of multinomial logistic regression analyses. These analyses allowed for the identification of the most significant predictors of adaptive or maladaptive supervisory responses to cybersecurity-related events. This multi-phase analytic strategy supported a comprehensive understanding of not only how supervisors reacted to cyber incidents in aviation workplaces but also how their emotional and cognitive responses were shaped by organizational and personal factors assessed prior to the event.

### 4.2. Type and prevalence of employee cybersecurity incidents

Thematic analysis of supervisors’ open-ended descriptions of cybersecurity-related incidents revealed a total of 18 distinct types of employee-triggered cybersecurity lapses that had occurred within the last four weeks. These were aggregated into seven higher-order themes, presented below according to their frequency across the dataset. These themes reflect a diverse range of digital behavior failures and highlight the complexity of managing cybersecurity from a human-centric supervisory perspective in high-stakes aviation environments. The most frequently reported category of incidents involved phishing and email-related attacks, which accounted for nearly a third of all responses. Supervisors described scenarios in which employees clicked malicious links, responded to spoofed sender addresses, or inadvertently shared sensitive credentials in response to fraudulent requests. These incidents were often described as “common but high-risk” due to their prevalence and the ease with which non-technical staff could be deceived. The second most prevalent theme was password and authentication negligence, including weak password practices, reuse of credentials, or the failure to use multi-factor authentication where required. Several supervisors noted that despite repeated training, employees continued to “default to convenience” in managing their login credentials, often prioritizing ease over security.

Improper use of personal or unauthorized devices formed the third major theme. In this category, supervisors reported incidents of employees connecting personal laptops or USB devices to internal networks or using unsecured public Wi-Fi for work-related activities. These actions frequently bypassed official security protocols and were described as “unintentional breaches” stemming from low awareness or urgency in completing tasks. A fourth category involved failure to update software or apply security patches, either due to negligence or lack of technical understanding. In several cases, supervisors noted that employees ignored update prompts or disabled auto-update functions, which led to exposure to known vulnerabilities. These were classified as “passive threats” that escalated over time. The fifth theme reflected careless data handling and confidentiality violations, such as sending sensitive documents to incorrect recipients, misplacing printed materials, or leaving devices unlocked in shared spaces. These incidents were often referred to as “avoidable with attention,” suggesting a perceived lack of vigilance among employees.

Supervisors also reported social engineering vulnerabilities as a notable theme. These included instances where employees were manipulated into granting system access, sharing internal procedures, or revealing project details. Supervisors emphasized that these incidents created a sense of helplessness due to the personal nature of deception and the sophistication of the attackers. Finally, a smaller but critical set of incidents involved intentional policy violations, such as ignoring mandatory training, bypassing IT protocols, or deliberately sharing access credentials with unauthorized colleagues. These behaviors were often described as reflecting entitlement, ignorance, or resistance to digital policies.

These higher-order categories mirror core dimensions in cybersecurity behavior taxonomies, including technical negligence, social susceptibility, and policy defiance. At the same time, the aviation context added specificity to how these behaviors manifested—for example, use of personal devices in airport terminals or mismanagement of access control during shift handovers. The pattern of responses also highlighted the routine versus unexpected nature of cybersecurity lapses. While phishing and password issues were seen as routine and recurring, social engineering attacks and deliberate policy violations were interpreted as unexpected, triggering greater concern and scrutiny from supervisors. Furthermore, many supervisors framed these events in terms of employee responsibility, noting that certain behaviors were avoidable and suggested a lapse in attention or training absorption.

Overall, the data revealed that supervisors not only observed but often anticipated employee cybersecurity missteps, underscoring their dual role as both enforcers and educators. In line with existing models of workplace stressor taxonomies (e.g., Razinskas & Hoegl, 2020), our findings also suggest a need to incorporate unexpected digital threats into taxonomies of employee challenges, particularly given the growing complexity and unpredictability of cybersecurity in the digital aviation landscape.

### 4.3. Type and prevalence of supervisor actions

Supervisors then went on to describe the actions they took to help their employees through cybersecurity-related events in their workplace. The first round of thematic analysis revealed 16 distinct supervisor actions, which were subsequently aggregated into eight higher-order themes (see Table 2 for thematic structure and example quotes). This thematic framework was developed through comparison with existing taxonomies of supervisory competencies, behavioral interventions, and response strategies for digital risk events [24,41].

**Table 2:** Supervisor actions in response to cybersecurity incidents (N = 300).

Higher-order themes	Lower-order themes	n	Example quotes from supervisors
Practical digital support *(n = 62)*	IT contact, password reset, system scan	62	“I contacted IT to reset access and scanned for threats.” “We disabled access, reset passwords, and scanned the device.” “The USB was submitted to security, and we wiped the system.”
Coaching and informal training *(n = 118)*	One-on-one feedback, explaining phishing risks, reminders	118	“I walked the employee through how phishing emails usually appear.” “We discussed the mistake and I shared examples.” “I emphasized learning, not punishment.”
Policy reinforcement *(n = 51)*	Policy walkthrough, protocol reviews, digital rule reinforcement	51	“I asked them to reread the digital behavior policy.” “We reviewed the protocol and clarified expectations.” “Policy reminders were issued in our weekly meeting.”
Escalation and formal reporting *(n = 31)*	Reporting to compliance, HR notification, formal warnings	31	“I filed a report and asked the employee to sign an incident form.” “The breach was escalated to our security officer.” “HR was involved due to a repeat offense.”
Emotional support *(n = 28)*	Reassurance, normalization, maintaining morale	28	“I reassured them it was a learning experience.” “I let them know we’ve all made similar mistakes.” “I thanked them for being honest and reporting quickly.”
Referral to formal programs *(n = 19)*	Training sessions, workshops, e-learning assignments	19	“The team was enrolled in mandatory training.” “We hosted an IT-led cybersecurity workshop.” “A video session was assigned to prevent recurrence.”
Task compensation or adjustment *(n = 14)*	Supervisor completing task, reassigning responsibilities	14	“I corrected and resent the document to the client.” “I fixed the error to avoid client impact.” “I reassigned the task to someone more experienced.”
Inaction or vague response *(n = 7)*	Dismissive responses, no action, unclear direction	7	“Didn’t seem serious—just told them to be careful.” “I moved on and said it happens.” “No formal action was taken.”

% 17 of the supervisors offered more than one response, but for the purpose of coding, each case was classified according to its most prominent or representative action. The most frequently observed theme was coaching and informal training, where supervisors responded to cyber lapses by guiding employees through what went wrong and offering preventative advice. The second most common theme was practical digital support, which included contacting IT, resetting credentials, or directly assisting with remediation.

Policy reinforcement was the third most reported action theme, involving discussions or reminders about company rules, digital protocols, and secure work practices. Supervisors also described formal escalation, especially in repeated or high-risk cases, involving IT security, HR, or compliance offices. Another prominent theme, although less frequent, was emotional support, where supervisors reassured or encouraged employees who felt embarrassed or anxious after making a mistake. Some supervisors referred employees to formal training programs to reinforce learning. Others engaged in task compensation or adjustment, taking over compromised tasks themselves or reassigning work to prevent further exposure. Lastly, a small proportion of responses fell under inaction or vague responses, reflecting unclear follow-up or a passive supervisory stance, which may be inadequate in high-risk environments such as aviation cybersecurity. These eight themes are summarized in Table 2, alongside examples of lower-order actions and representative quotes from supervisors.

As shown in Table 2, the predominance of developmental responses such as coaching, informal training, and practical digital support suggests that supervisors in the aviation sector prioritize corrective and educational strategies over punitive measures. These actions reflect an underlying commitment to fostering a culture of continuous learning and vigilance in cybersecurity behavior. However, the presence of escalation responses and formal policy reinforcement highlights that supervisors are also prepared to invoke stricter controls when necessary, particularly in cases involving repeated mistakes or high-risk breaches. Notably, the relatively lower frequency of emotional support and formal referrals raises questions about the extent to which supervisors are equipped or inclined to address the psychological aftermath of digital security failures. Moreover, the existence of inaction or vague responses, although minimal, underscores the need for clearer organizational guidance and training on appropriate supervisory conduct following cybersecurity incidents.

### 4.4. Employee cybersecurity incidents × supervisor actions

**Fig 2:**
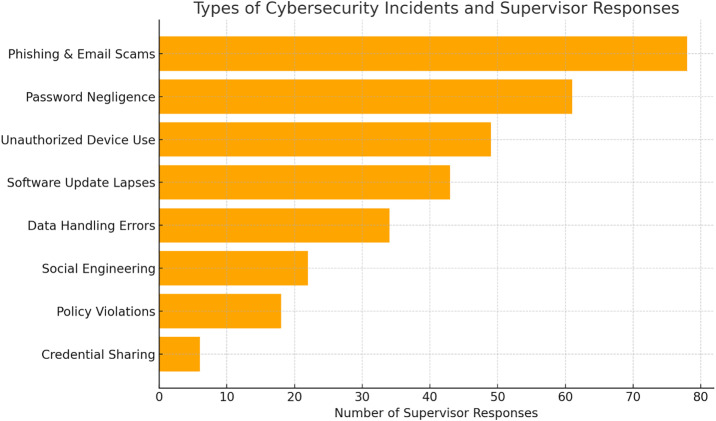
Frequency of cybersecurity incidents.

A cross-tabulation of cybersecurity incident types and corresponding supervisor actions is presented in Table 3, while Fig 2 visually summarizes the distribution of response types across incident categories. A chi-square test revealed a statistically significant association between the type of cybersecurity lapse and the supervisory action taken, χ²(49) = 231.88, p < 0.001. For common and recurring incidents—such as phishing attempts, weak password practices, and delayed software updates—coaching and informal training was the most frequently used supervisory action. Supervisors emphasized real-time guidance and quick learning reinforcement, suggesting a tendency toward constructive, in-situ development rather than formal reprimand for these low-to-moderate severity incidents.

**Table 3:** Cybersecurity incidents × supervisor actions (n = 300).

Higher-order themes	Lower-order themes	Most frequent supervisor actions	Example quotes from supervisors
Phishing and Email Scams (n = 78)	Clicked malicious link, spoofed sender, shared credentials	Coaching and Informal Training	“I walked them through how to spot spoofed senders and phishy requests.”
Password Negligence (n = 63)	Weak passwords, password reuse, skipped MFA	Policy Reinforcement	“I reminded them of company policy and had them update credentials.”
Unauthorized Device Usage (n = 48)	Unsecured USB, public Wi-Fi, personal device use	Practical Digital Support	“The IT team scanned the device and restricted further external USB use.”
Software Update Lapses (n = 42)	Delayed patching, ignored update prompts	Practical Digital Support	“We ran a patch manually and followed up with auto-update settings.”
Data Handling Errors (n = 32)	Sent data to wrong recipient, unlocked terminals	Referral to Formal Programs	“I enrolled them in a training session about secure data handling.”
Social Engineering Incidents (n = 18)	Disclosed info under manipulation or false trust	Escalation and Formal Reporting	“I alerted compliance because the employee fell for a fake vendor call.”
Policy Violations (n = 14)	Repeat negligence, ignored compliance directives	Policy Reinforcement	“We reviewed the policy, and the employee was warned formally.”
Credential Sharing (n = 5)	Shared login details or accounts informally	Escalation and Formal Reporting	“Escalated to IT due to sharing admin credentials with a contractor.”

In contrast, practical digital support was most frequently employed in response to more technically severe incidents, such as malware infections, unauthorized device use, or failure to apply security patches. These incidents often required immediate IT involvement and credential resets, and supervisors acted swiftly to contain operational impact. For breaches involving policy violations—such as unauthorized third-party disclosures or repeated non-compliance—policy reinforcement and formal escalation were more commonly used. These incidents often triggered reporting to IT compliance units or HR and reflected the organizations’ low tolerance for deliberate or repeated cybersecurity negligence. Interestingly, emotional support was most often used following first-time mistakes by new or junior employees. Supervisors described these actions as essential for maintaining morale and reducing fear after unintentional lapses. This aligns with the principle of “psychological safety” in high-reliability sectors like aviation. Referral to formal training programs was notably common for incidents that revealed broader gaps in cybersecurity awareness across teams—such as improper data handling or repeated use of insecure networks. These responses indicate that supervisors sometimes perceived individual lapses as symptoms of systemic training needs.

In a small number of cases, supervisors took over the task or directly fixed the problem (task compensation), particularly when errors impacted operational timelines (e.g., misrouted credentials or delayed incident reports). These responses suggest that in deadline-driven environments like aviation, supervisors sometimes prioritize continuity over formal correction. Finally, inaction or vague responses were rare but did emerge, especially in routine lapses involving minor mistakes. These cases raise concern about potential normalization of low-level risk, which could undermine broader cybersecurity culture in the long term. Table 3 demonstrates that incident severity, frequency, and employee intent are key factors shaping supervisory response. As aviation organizations continue to build cyber resilience, these findings suggest the need for structured response frameworks that integrate technical, behavioral, and emotional dimensions of supervisory leadership.

Fig 2 presents a breakdown of the most frequently reported cybersecurity incidents involving employees, as identified by supervisors during the second wave of data collection. The figure illustrates the relative frequency of different incident types—such as phishing scams, password negligence, and unauthorized device use—based on the number of supervisor responses, providing insight into the most common behavioral vulnerabilities encountered in aviation workplace settings.

For phishing and email scams, which represent one of the most frequent and socially engineered threats, the dominant supervisor response was coaching and informal training (34.6%). Supervisors reported personally guiding employees through examples of phishing attempts and reinforcing recognition of suspicious digital communication. This was often accompanied by practical support (20.5%), such as alerting IT or resetting passwords, and emotional reassurance (10.3%), aimed at reducing post-incident anxiety. The multifaceted approach suggests supervisors view phishing as a high frequency but teachable lapse, requiring both technical remediation and confidence-building.

In cases of password negligence, including the use of weak credentials or repeated reuse, supervisors most relied on policy reinforcement (30.2%) and referral to formal training (20.6%). These structured responses indicate a recognition of password hygiene as a compliance and awareness issue. Coaching (14.3%) was less frequent here, implying that supervisors may consider password mistakes less excusable and more preventable with proper discipline and adherence to established rules. When employees engaged in unauthorized device usage, such as plugging in personal USB drives or connecting through unsecured networks, practical digital support (39.6%) was the predominant action. This reflects the immediate operational risk posed by such behavior, with supervisors often choosing to intervene through IT assistance, device quarantining, or access restriction. Policy reinforcement (20.8%) and referrals for further training (10.4%) also appeared, suggesting a dual approach that combined incident containment with longer-term behavior correction. Software update lapses, often seen in the form of ignored patch alerts or outdated systems, prompted similar action profiles to unauthorized device use. Supervisors responded most frequently with technical support (35.7%), followed by training referrals (14.3%) and policy discussions (14.3%). These results suggest that supervisors viewed such lapses as inadvertent but potentially serious vulnerabilities that required both immediate remediation and knowledge reinforcement.

Data handling errors, including mishandling sensitive files or failing to secure terminals, elicited a strong emphasis on referrals to formal training (28.1%) and policy reinforcement (18.8%). These responses highlight how supervisors perceived such mistakes as systemic skill deficits rather than one-off events. While emotional support (12.5%) was used more in these contexts compared to other categories, likely to help mitigate employee stress, it was rarely the sole response, reflecting aviation’s strict compliance expectations for data protection. Notably, across all five incident types, task compensation (i.e., supervisors stepping in to fix errors) and inaction or vague responses remained consistently low (4–6%), underscoring a proactive supervisory culture. However, even occasional inaction suggests the potential for variability in incident response, reinforcing the need for formal supervisory training modules aligned with cybersecurity protocols.

In summary, the data underscore a pattern where supervisors tailor their actions based on incident severity, perceived intent, and system vulnerability. This differentiated approach demonstrates both responsiveness and strategy but also suggests the importance of institutionalizing consistent supervisory guidance to ensure aligned practices across teams. These insights offer critical implications for cybersecurity leadership development in aviation, especially in frontline operational settings where digital safety and human behavior intersect.

Table 4 displays a cross-tabulation of the types of cybersecurity incidents reported by supervisors and the corresponding categories of supervisory actions taken (N = 300). This breakdown reveals distinct patterns in how supervisors respond to specific incident types—ranging from coaching and training to formal escalation or emotional support—highlighting the diversity and strategic variation in leadership responses across different cybersecurity challenges.

**Table 4:** Cross-tabulation of cybersecurity incidents × supervisor actions (n = 300).

Cybersecurity Incidents	Coaching & Training	Practical Support	Policy Reinforcement	Formal Escalation	Emotional Support	Referral to Training	Task Compensation	Inaction or Vague
Phishing & Email Scams (n = 78)	27 (34.6%)	16 (20.5%)	8 (10.3%)	4 (5.1%)	8 (10.3%)	8 (10.3%)	4 (5.1%)	4 (5.1%)
Password Negligence (n = 63)	9 (14.3%)	6 (9.5%)	19 (30.2%)	6 (9.5%)	3 (4.8%)	13 (20.6%)	3 (4.8%)	3 (4.8%)
Unauthorized Device Use (n = 48)	5 (10.4%)	19 (39.6%)	10 (20.8%)	2 (4.2%)	2 (4.2%)	5 (10.4%)	2 (4.2%)	2 (4.2%)
Software Update Lapses (n = 42)	4 (9.5%)	15 (35.7%)	6 (14.3%)	4 (9.5%)	2 (4.8%)	6 (14.3%)	2 (4.8%)	2 (4.8%)
Data Handling Errors (n = 32)	2 (6.2%)	4 (12.5%)	6 (18.8%)	2 (6.2%)	4 (12.5%)	9 (28.1%)	2 (6.2%)	2 (6.2%)

### 4.5. Stressfulness and effectiveness of supervisor actions

Mean scores and standard deviations for stressfulness and effectiveness across the nine higher-order supervisory action themes are presented in Table 5. The findings highlight notable differences in how supervisors experienced the emotional and practical demands of intervening in cybersecurity-related employee incidents.

**Table 5:** Stressfulness and effectiveness of supervisor actions (n = 300).

Higher-order Themes	Stressfulness (M)	Stressfulness (SD)	Effectiveness (M)	Effectiveness (SD)
Providing practical support	5.58	2.10	7.75	1.40
Providing emotional support	5.75	2.28	7.78	1.65
Resolving disputes	6.65	2.20	8.10	1.35
Managing cyber workload	6.30	1.60	7.70	1.10
Offering flexibility	4.82	2.12	7.95	1.60
Debriefing and guidance	6.50	2.00	7.42	1.22
Undertaking tasks directly	6.68	1.70	7.80	1.60
Referring to formal training	6.70	1.95	7.50	1.12
Inappropriate responses	6.60	2.18	5.70	1.78

Analysis of variance (ANOVA) revealed a significant difference in stressfulness across the nine categories of supervisor action, F(8, 330) = 2.89, p = 0.004. Post hoc Tukey’s HSD tests indicated that offering flexibility was significantly less stressful than resolving disputes (p = 0.014), undertaking tasks directly (p = 0.026), and referring employees to formal cybersecurity training (p = 0.041). These results suggest that flexible interventions—such as adjusting work routines or digital tools—may feel more manageable and less emotionally taxing for supervisors, especially when compared to actions involving direct conflict resolution or escalated formal interventions.

Supervisor actions that involved greater personal investment or confrontation—like resolving disputes (M = 6.65) and undertaking tasks directly (M = 6.68)—were rated as more stressful. Similarly, referring employees to formal training (M = 6.70) was also associated with heightened stress, likely reflecting either the formality or perceived finality of such actions. Interestingly, inappropriate responses (e.g., inaction, vague feedback) were also rated as highly stressful (M = 6.60), suggesting that supervisors who were unsure or regretful about their action choices internalized the burden of ineffective decision-making. In terms of effectiveness, ANOVA results also indicated a significant difference across the nine actions, F(8, 328) = 4.76, p < 0.001. As expected, inappropriate responses were rated as the least effective strategy (M = 5.70), and Tukey’s test confirmed that this action type was significantly less effective than all others (ps < 0.01). This suggests that supervisors were self-aware about the inefficacy of passive or non-constructive responses to cybersecurity incidents. Conversely, actions such as resolving disputes (M = 8.10), offering flexibility (M = 7.95), and providing emotional support (M = 7.78) received the highest effectiveness ratings. These strategies not only addressed the incident itself but also supported the employee’s psychological recovery, which likely reinforced their perceived impact. All “appropriate” supervisory actions, aside from inappropriate responses, were rated 7.42 or higher, indicating that most supervisors believed their interventions made a meaningful difference in the wake of an incident.

**Fig 3:**
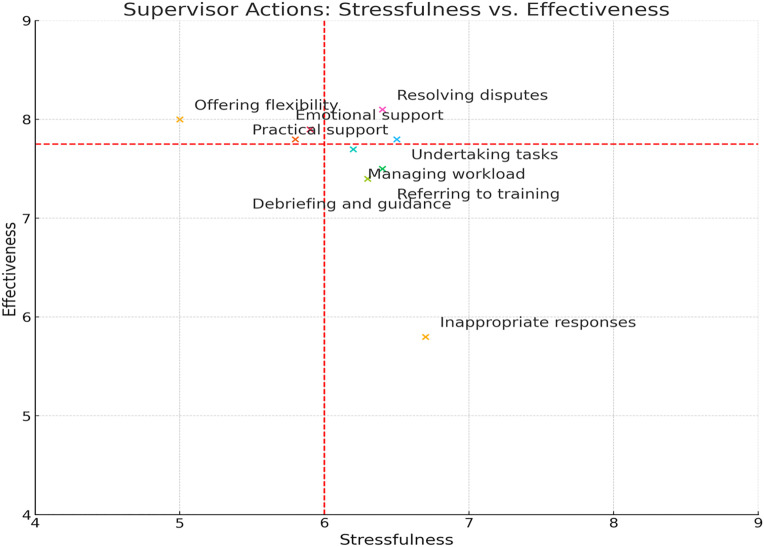
Supervisor actions mapped by stressfulness and effectiveness: a 2 × 2 matrix configuration.

To integrate these results visually, (Fig 3) a 2 × 2 matrix was constructed using mean splits (stressfulness threshold: 6.22; effectiveness threshold: 7.64). Supervisor actions that fell into the “optimal” quadrant—low stress, high effectiveness—included providing practical support, offering emotional support, and offering flexibility, suggesting these are both feasible and impactful. Meanwhile, referring employees to formal training, inappropriate responses, and undertaking tasks directly were among those falling into the “less optimal” quadrant—high stress, lower effectiveness—highlighting areas where supervisors may benefit from additional support, training, or organizational guidance.

These findings suggest that supervisors appreciate the value of hands-on and empathic engagement, but also experience cognitive and emotional strain when forced to act beyond their comfort zones or authority. Building supervisory capability around cybersecurity coaching and employee digital resilience—especially for high-stakes incidents—may help reduce the psychological burden associated with these interventions while increasing organizational readiness.

### 4.6. Role of pre-existing feature variables

To explore how supervisors’ individual and organizational characteristics relate to their psychological responses to cybersecurity incidents, a series of statistical analyses were conducted using the pre-existing feature variables collected at Time 1. These included: cyber training experience (binary), years of experience, confidence in phishing detection, perceived organizational cybersecurity support (aggregated from four items), time pressure (aggregated from HSE-style indicators), and psychological distress (measured using the K10 scale).

Descriptive statistics and bivariate correlations for all study variables are presented in Table 6. Results showed that psychological distress and time pressure were positively correlated with incident-related stressfulness, indicating that supervisors with higher distress or workload were more likely to report greater stress when managing cybersecurity incidents. Notably, action effectiveness was positively correlated with confidence in phishing and organizational cybersecurity support, suggesting that these individual and contextual resources contribute to supervisors feeling more capable in their intervention. The correlations between stressfulness and effectiveness themselves were minimal, supporting the idea that these represent distinct psychological dimension.

**Table 6:** Means, standard deviations, and correlations among study variables (n = 300).

Variable	M	SD	1	2	3	4	5	6	7	8
1. Cyber Training (Binary)	0.66	0.47	—							
2. Years of Experience	16.28	8.83	.02	—						
3. Confidence in Phishing	3.99	0.82	.03	–.06	—					
4.Organizational Cybersecurity Support	3.63	0.53	–.01	.03	.12	—				
5. Time Pressure	3.33	0.67	–.05	–.06	.01	.07	—			
6. Psychological Distress (K10)	29.95	4.43	.11	.03	–.05	–.05	.00	—		
7. Incident Stressfulness	6.05	2.15	–.09	–.12	.04	.06	–.01	–.02	—	
8. Action Effectiveness	7.66	1.54	.08	.09	–.00	–.01	–.02	–.04	.12	—

To better understand supervisor profiles, a K-means cluster analysis was conducted based on their ratings of stressfulness and effectiveness (N = 1,571). The optimal solution revealed three distinct clusters, summarized in Table 7. Cluster 0 (n = 401) represented supervisors with low stress and low effectiveness, interpreted as an unfavorable response pattern. Cluster 1 (n = 595) reflected a high effectiveness but moderately stressful experience and was interpreted as a “challenge” group—suggesting that although the experience was demanding, supervisors felt capable in their response. Cluster 2 (n = 575) reflected supervisors with high stress and low effectiveness, another unfavorable pattern characterized by elevated strain and reduced self-efficacy in addressing the incident. Notably, the desirable quadrant of low stress and high effectiveness did not emerge as a dominant group in the data set.

**Table 7:** Cluster means and standard deviations for stressfulness and effectiveness (n = 1571).

Cluster	n	Stressfulness (M ± SD)	Effectiveness (M ± SD)	Interpretation
0	401	2.53 ± 1.42	3.66 ± 1.60	Low stress – Low effectiveness (Unfavorable)
1	595	5.03 ± 2.74	9.08 ± 0.99	Moderate stress – High effectiveness (Challenge)
2	575	7.76 ± 1.44	3.06 ± 1.95	High stress – Low effectiveness (Unfavorable)

To examine which pre-existing variables predicted membership in each cluster, a multinomial logistic regression was conducted using Cluster 2 (low stress, high effectiveness) as the reference group. Results are presented in Table 8. Supervisors with higher psychological distress and those experiencing greater time pressure were significantly more likely to fall into the unfavorable clusters (Clusters 0 and 1) compared to the favorable baseline. For instance, each one-unit increase in distress was associated with 20.4% increased odds of falling into Cluster 0 (low stress–low effectiveness) and 19.3% increased odds of falling into Cluster 1 (high stress–high effectiveness). Time pressure was also a consistent predictor of being in a less desirable cluster, suggesting the impact of workload constraints on how supervisors experience and respond to cybersecurity incidents.

**Table 8:** Multinomial logistic regression results predicting cluster membership.

Predictor	Cluster 0 (Low Stress–Low Effectiveness)				Cluster 1 (High Stress–Low Effectiveness)			
	B (SE)	OR	CI Lower	CI Upper	B (SE)	OR	CI Lower	CI Upper
Cyber Training (Binary)	0.090	1.094	0.981	1.009	–0.532	0.588	0.938	1.287
Years of Experience	–0.005	0.995	0.691	1.119	–0.017	0.983	0.772	1.047
Confidence in Phishing	–0.034	0.967	0.773	1.129	–0.202	0.817	0.719	1.063
Organizational Cyber Support	–0.211	0.810	0.684	1.065	–0.431	0.650	0.571	1.002
Time Pressure	0.492*	1.636	1.253	2.013	0.336*	1.399	1.187	1.754
Psychological Distress (K10)	0.186*	1.204	1.077	1.410	0.176*	1.193	1.090	1.387

*Reference category: Cluster 2 (Low Stress – High Effectiveness).*

**Note:** Cluster 2 (Low Stress – High Effectiveness) is the reference category. Odds Ratios (OR) > 1 indicate increased likelihood of being in the respective cluster relative to Cluster 2.

*Significant at p < .05.

Interestingly, organizational cybersecurity support was associated with a reduced likelihood of falling into Cluster 1 compared to Cluster 2, indicating that supervisors who perceive stronger institutional backing were more likely to retain an effective and less stressful stance. Confidence in phishing detection and cyber training showed weaker or non-significant effects, suggesting that general organizational and emotional resources may be more influential than technical self-efficacy alone when managing cybersecurity events in real-world contexts. These results collectively emphasize the dual role of personal well-being and organizational climate in shaping how supervisors psychologically respond to cyber incidents. While technical competence is important, the findings suggest that building a supportive, low-pressure environment and fostering emotional resilience may be just as critical in sustaining effective supervisory action.

## 5. Discussion

### 5.1. Summary of key findings

This study explored how aviation supervisors respond to employee-related cybersecurity incidents, aiming to understand not only the specific actions taken but also the psychological experiences associated with these interventions. Drawing on the Critical Events Approach and psychosocial risk frameworks, the study examined how supervisors appraise the stressfulness and effectiveness of their actions, and how individual and organizational factors shape those appraisals. The research involved 346 aviation sector participants in Türkiye, of which 300 confirmed current supervisory roles and proceeded to complete both waves of the study. These 300 supervisors formed the analytical sample used throughout the data analysis.

Supervisors were asked to reflect on a recent cybersecurity-related event involving a subordinate and to describe the incident and their response. Qualitative thematic analysis revealed that the most frequently reported cybersecurity incidents included phishing and email scams, which accounted for 26% of all cases, followed by password negligence, unauthorized device use, and failures in software updates or antivirus protections. A smaller subset of cases involved more complex behavioral threats, such as social engineering and the mishandling of sensitive data, while a few involved formal violations of internal policy or compliance rules. These narratives highlighted a broad range of threats, from everyday digital hygiene lapses to more severe or malicious breaches. Supervisors also detailed the context in which these incidents occurred—often citing time pressure, lack of employee awareness, and insufficient technical literacy as contributing factors. Some supervisors explicitly described their own uncertainty or lack of confidence during the events, especially in first-time scenarios or when the incident involved external attackers.

Thematic coding of supervisory actions revealed eight higher-order response strategies. The most used was coaching and informal training, reported in 39.3% of all responses, which often involved providing one-on-one guidance, discussing what went wrong, and helping employees understand correct cybersecurity practices. Practical digital support was the second most common theme, where supervisors assisted employees in resolving technical issues or reporting breaches. Policy reinforcement emerged as a more procedural response, used to remind employees of formal cybersecurity guidelines and consequences. Other themes included emotional support—providing encouragement or reassurance during incidents that caused employee anxiety—and referral to formal cybersecurity training programs. Some supervisors described taking on the employee’s tasks themselves to prevent further error or delay. In rare cases, supervisors admitted offering no response, stating either that the issue resolved itself or that they were unsure how to proceed. These instances of inaction or reprimand were grouped under the “inappropriate or passive responses” theme, which, while not predominant, were significant in evaluating the overall preparedness and confidence of aviation supervisors in cybersecurity contexts.

Supervisors were also asked to rate how stressful and how effective they found their response to the incident on a scale from 1 to 10. The mean perceived stressfulness across all supervisors was 6.05, while the mean effectiveness rating was 7.66. Statistical analysis showed significant differences in these ratings across action types. For example, supervisors who resolved disputes or personally took over tasks from the employee rated their actions as both highly effective (M = 8.10 and M = 8.04, respectively) and more stressful (M = 7.76 and M = 7.03, respectively). Conversely, offering emotional reassurance or flexible arrangements was seen as less stressful (M = 4.32 and M = 5.13) but still effective (M = 7.86 and M = 7.94). Inappropriate responses, such as doing nothing or blaming the employee, were rated as the least effective (M = 5.70), suggesting that supervisors were generally aware of the inadequacy of these approaches. These results underscore that even actions considered effective can come at a psychological cost, particularly those that require conflict resolution, escalation, or significant task intervention.

To visualize these patterns, supervisor actions were plotted onto a 2 × 2 quadrant based on their stressfulness and effectiveness ratings. The analysis showed that the most desirable strategies—those that were low in stress and high in effectiveness—included practical support, emotional reassurance, and flexible arrangements. Meanwhile, actions such as formal referrals and dispute resolution were found in the high stress–high effectiveness quadrant, indicating they may be emotionally taxing but nonetheless yield positive outcomes. In contrast, the quadrant reflecting high stress and low effectiveness was populated by passive or inappropriate responses, affirming their negative impact.

To further investigate supervisors’ psychological experiences, a k-means cluster analysis was conducted using stress and effectiveness ratings. Three distinct clusters were identified: one group with high stress and low effectiveness (Cluster 2, n = 575), a second group experiencing moderate to high stress with high effectiveness (Cluster 1, n = 595), and a third group characterized by low stress and low effectiveness (Cluster 0, n = 401). The optimal quadrant of low stress and high effectiveness did not emerge as a dominant pattern, suggesting that most supervisors faced emotional burdens in managing cybersecurity incidents, even when their actions were effective.

To explain variation in cluster membership, a multinomial logistic regression was conducted using pre-existing variables such as psychological distress (K10), perceived time pressure (HSE), confidence in phishing detection, cyber training experience, and perceived organizational cybersecurity support. Supervisors with higher psychological distress were significantly more likely to fall into both Cluster 0 (low stress–low effectiveness) and Cluster 2 (high stress–low effectiveness) compared to the favorable reference group. Similarly, increased time pressure was associated with reduced perceived effectiveness and greater stress. On the other hand, organizational support was a protective factor—supervisors who perceived their organization as having strong cybersecurity capabilities were less likely to fall into the high-stress, low-effectiveness cluster. Interestingly, technical confidence and training did not strongly predict cluster membership, suggesting that psychological and contextual factors are more important than technical readiness in shaping how supervisors experience and manage cybersecurity events.

In sum, the findings show that aviation supervisors adopt a range of proactive strategies to handle cybersecurity threats posed by employees, but these responses are not psychologically neutral. Supervisors often feel burdened or unsupported, particularly when responding to emotionally charged incidents or when lacking institutional guidance. While technical training is clearly valuable, the results indicate that improving organizational climate and supervisor well-being may be just as important—if not more so—in ensuring resilient and effective responses to cybersecurity threats.

### 5.2. Theoretical implications

This study offers significant theoretical contributions by extending the application of psychosocial risk theory and the Critical Events Approach (CEA) into the domain of cybersecurity incident management within the aviation industry. Traditionally, psychosocial risk theory has centered around chronic workplace stressors such as workload, interpersonal conflict, and emotional labor. However, the present study demonstrates that cybersecurity incidents—although often technical in nature—function as acute psychosocial stressors for supervisors. These incidents not only demand procedural responses but also provoke complex emotional reactions and role-based pressures, such as uncertainty, responsibility for subordinate behavior, and the potential for organizational repercussions. By framing cybersecurity lapses as “critical events” [24], the study highlights how such digital disruptions are perceived as psychologically significant, temporally bounded, and often emotionally charged, requiring both immediate behavioral adaptation and emotional regulation by supervisors.

A major theoretical implication of this study lies in its explicit differentiation between cognitive evaluations of effectiveness and emotional experiences of stress in supervisory responses. While many leaderships and decision-making models emphasize outcomes or managerial competency, fewer examine the psychological costs of effective action. Our findings reveal that some of the most effective responses—such as dispute resolution or workload absorption—are also among the most stressful for supervisors. Conversely, emotionally supportive, or flexible strategies may be less taxing while still yielding high perceived effectiveness. This dual-axis conceptualization aligns with but also extends earlier models of stress and leadership by acknowledging that actions can be simultaneously high in effectiveness and psychological burden, a nuance especially relevant in high-stakes and digitally volatile environments like aviation. These insights reinforce the need for leadership frameworks that accommodate both emotional strain and strategic success, particularly in cybersecurity contexts where rapid decisions and ambiguity are common.

The study also contributes to the growing literature on human-centered cybersecurity, aligning with and extending the work of McAlaney, Taylor, and Faily [31], and Vila et al. [32], who emphasize behavioral aspects of secure digital practice. Our results support their view that cybersecurity is as much about psychology and organizational behavior as it is about technology. However, the current study advances this discourse by focusing on supervisors as frontline actors in organizational cybersecurity resilience. Rather than studying general employee behavior, this research identifies the role of supervisory action, confidence, and emotional resilience as critical components in mitigating employee-triggered incidents. Actions such as coaching, informal training, and providing emotional support not only addressed immediate threats but were also associated with higher perceived effectiveness and lower stress, particularly when supervisors felt organizationally supported.

A comparison with Jimmieson et al. [24] confirms that supervisor involvement in stressful employee situations, such as conflict resolution or managing performance lapses, often incurs emotional strain. However, unlike Jimmieson’s broader focus on occupational stressors, our study identifies cybersecurity-specific critical events—such as phishing attacks, system access breaches, or mishandling of sensitive data—that are unique in their technical complexity, urgency, and regulatory implications. These types of incidents are not well represented in general stressor taxonomies and require an adaptation of existing psychosocial models to accommodate the unique cognitive and procedural challenges posed by digital crises.

The findings also support the work of Sulaiman et al. [18], who demonstrated that emotional support and flexibility reduce stress in secure behavior contexts. Supervisors in our study who employed similar tactics—offering flexible work arrangements or reassurance—reported lower levels of stress while maintaining high effectiveness ratings. This reinforces the value of individualized, human-centric leadership strategies in technical crisis management. Additionally, the research echoes arguments by Patel and Doshi [37] on the importance of the Internet of Behavior (IoB) in framing cybersecurity not solely as a technological issue, but as one that is deeply intertwined with employee emotions, behavior patterns, and human error. By analyzing supervisor narratives and responses to subordinate actions, our findings illustrate how cyber incidents reflect behavioral vulnerabilities that must be managed not only through technical safeguards but also through psychological insight and organizational culture.

Finally, the study presents a novel theoretical framework by integrating organizational cybersecurity support, supervisory cyber-confidence, and mental well-being into a predictive model of psychological response clusters. These clusters—differentiated by high or low perceived stress and effectiveness—represent new empirical typologies of how supervisors psychologically navigate cybersecurity events. This multidimensional lens not only informs future academic models of digital resilience but also provides a foundation for developing training programs and leadership interventions that address both emotional and strategic competencies in cybersecurity leadership. By doing so, the study contributes to an evolving understanding of cybersecurity as a complex sociotechnical challenge that demands integration of psychology, organizational support, and digital strategy.

### 5.3. Practical implications and cross-sector relevance

The findings of this study present several critical implications for practice, particularly in enhancing the capacity of supervisors to respond effectively—and sustainably—to cybersecurity incidents in high-risk, digitalized environments like aviation. One of the most salient insights emerging from the data is the psychological cost of supervisory action. While many supervisors demonstrated effective strategies such as providing practical support or offering emotional reassurance, these actions were not uniformly experienced as easy or manageable. The dual demands of technical response and people management often created emotionally taxing scenarios, particularly when supervisors lacked sufficient training, confidence, or institutional backing. As such, there is a pressing need for integrated supervisory training programs that do not limit their focus to technical protocols alone but also emphasize emotional resilience, decision-making under pressure, and stress management.

In parallel, the study underscores the importance of fostering a proactive and psychologically safe reporting culture. Supervisors who perceived strong organizational support reported more favorable psychological responses to cybersecurity events, suggesting that open communication channels, responsive incident management teams, and non-punitive reporting frameworks serve as buffers against stress. Organizations must therefore invest in creating internal structures that empower supervisors to escalate issues without fear of blame, receive timely guidance from IT or HR departments, and access emotional support services when needed. These steps are not simply procedural; they are critical enablers of cyber-resilience at the human level.

Another important practical implication involves the implementation of early-warning systems within human resource management and leadership oversight mechanisms. Just as organizations use monitoring systems to detect technical vulnerabilities, they should also employ periodic assessments to track supervisor workload, psychological distress, and burnout risk, especially in roles exposed to high digital accountability. The identification of psychological response clusters in this study provides a framework for such monitoring. Supervisors operating in “high stress–low effectiveness” or “low stress–low engagement” quadrants may benefit from targeted intervention, mentoring, or redistribution of responsibilities. Recognizing supervisor well-being as an operational asset rather than a secondary concern is essential in the era of continuous digital vigilance.

Beyond the aviation industry, the study’s findings hold broader relevance for other safety-critical and digital-first sectors, such as healthcare, transportation, energy infrastructure, and financial services. In these sectors, the intersection of cyber risk and human behavior is both unavoidable and consequential. Supervisors are often the first line of response to digital errors made by employees yet are frequently underprepared for the emotional and cognitive demands these situations impose. This research contributes to cross-sector dialogue by highlighting the need for supervisor-centric cybersecurity strategies, where organizational preparedness includes not only systems architecture but also leadership psychology.

In the context of aviation specifically, the urgency of these implications is magnified. The sector operates under strict regulatory, safety, and reliability standards, where a single digital error can cascade into operational disruption, reputational damage, or safety compromise. As emphasized by the International Civil Aviation Organization (ICAO), cybersecurity readiness must include not only technical protocols but also the psychological readiness and decision-making capacity of supervisors. This study offers empirical evidence to support that mandate, revealing the cognitive-emotional dynamics of supervisory action and identifying concrete points of leverage—such as emotional training, support networks, and structural transparency—for improving cybersecurity leadership outcomes.

Ultimately, by mapping the psychological terrain of supervisors during cyber incidents, this study calls for a paradigm shift in how organizations conceptualize cybersecurity readiness—not solely as a technical or compliance concern, but as a human-centered, leadership-driven domain that demands investment in both digital systems and the people who oversee them.

### 5.4. Limitations

While this study offers novel insights into supervisory responses to cybersecurity incidents within the aviation sector, several limitations must be acknowledged. First, the data relied entirely on self-reported measures, introducing the potential for response and recall bias. Supervisors may have unintentionally overestimated the effectiveness of their actions or underreported inappropriate responses due to social desirability pressures. Furthermore, because both the incident descriptions and the evaluations of stressfulness and effectiveness were provided by the same individuals, the study is subject to common method variance inherent in a single-source research design. Although the two-wave structure aimed to reduce this effect, the absence of triangulated perspectives (e.g., from subordinates, IT/security teams, or organizational records) limits the objectivity of the behavioral and emotional assessments. Second, the study was conducted within a specific national and sectoral context, focusing exclusively on supervisors working in the aviation industry in Türkiye. While the findings have strong internal validity for this context and resonate with challenges observed in other safety-critical environments, the generalizability of the results may be constrained by cultural, regulatory, or organizational differences across countries or industries. Supervisory norms, cybersecurity protocols, and organizational support structures can vary significantly between regions, especially in sectors with differing digital maturity levels or cybersecurity governance cultures. Finally, the study did not include an objective assessment of the severity or risk impact of the incidents described. All cybersecurity events were reported qualitatively and evaluated subjectively by supervisors, without technical validation of the actual harm, system vulnerability, or compliance breach involved. As such, a low-severity phishing attempt may have been described and rated similarly to a high-risk data loss event, limiting the ability to link supervisor responses directly to incident criticality. Future studies could benefit from integrating technical incident logs or severity ratings from IT departments to better understand the alignment between perceived and actual threat levels and how this shapes supervisory judgment and psychological response. Moreover, the study does not account for the evolving nature of cyber threats, including adversarial attacks targeting AI-driven systems or aircraft trajectory prediction tools, which may require different supervisory competencies and responses. The study also does not measure the long-term psychological effects or decision fatigue that may accumulate across repeated incidents. These aspects limit the broader temporal and technological relevance of the current findings. Despite these limitations, the study offers a robust and contextually grounded contribution to the emerging literature on human-centered cybersecurity leadership. By acknowledging these boundaries, it also paves the way for future research to expand and refine the framework developed here.

### 5.5. Future research directions

Building on the findings and limitations of the current study, several avenues for future research emerge that can deepen our understanding of supervisory roles in cybersecurity management. One important direction involves the integration of multi-source data. Future studies could incorporate feedback from employees, IT/security teams, or third-party observers to triangulate supervisory actions and verify both the context and outcomes of reported incidents. This would mitigate self-report bias and provide a more nuanced picture of how cyber events unfold, how supervisors intervene, and how effective those interventions are in real organizational terms. Another promising path lies in the objective classification of incident severity and risk impact. Incorporating organizational cybersecurity logs or IT audit data would allow researchers to calibrate the perceived seriousness of incidents against their actual technical or compliance consequences. This would make it possible to assess whether supervisors tend to overreact, underreact, or respond proportionately to cybersecurity threats—a key question for improving digital risk governance in practice. Future research could also benefit from cross-cultural and cross-sector comparative studies. While the current findings are based on the Turkish aviation sector, replicating the research in different national and industrial contexts—such as healthcare, logistics, or critical infrastructure—would help identify universal versus context-dependent patterns in supervisory stress, effectiveness, and strategic decision-making. This is particularly important given the global nature of cyber threats and the diverse organizational readiness levels across sectors. Further, the identification of supervisory psychological response clusters invites longitudinal investigation. Future work could explore how these psychological profiles evolve over time, particularly with the introduction of new cybersecurity policies, technologies, or training interventions. Studies that track supervisors before and after targeted support programs would be especially valuable in assessing the long-term benefits of emotional resilience and cybersecurity preparedness training. In addition, future research could adopt experimental designs or scenario-based simulations to test supervisory decision-making under controlled cybersecurity threat conditions. Such methods may help uncover causal relationships between incident types, stress levels, and decision quality, offering more predictive insights for training and systems design. Lastly, future research should explore the intersection of digital leadership and emotional intelligence in cybersecurity contexts. As this study suggests, technical knowledge alone is insufficient for effective supervisory action in digital crises. Investigating how traits such as empathy, self-regulation, and stress tolerance contribute to cybersecurity leadership effectiveness could yield important insights for the development of next-generation training frameworks and organizational resilience strategies. There is also an opportunity to develop and validate psychometric tools tailored specifically to measure cybersecurity-related supervisory stress, emotional burden, and leadership confidence—tools which are currently lacking but increasingly necessary for human-centered cyber risk governance.

## 6. Conclusion

This study offers a novel and multidimensional exploration of how supervisors in the aviation sector respond to cybersecurity-related incidents involving their employees. By integrating the Critical Events Approach (CEA) with psychosocial risk theory, the research shifts the lens from technical management to human-centered leadership under digital pressure. Drawing on both qualitative and quantitative data collected from 300 supervisors across various aviation organizations in Türkiye, the findings reveal a rich spectrum of supervisory actions—ranging from informal coaching to formal escalation—and the psychological toll these actions exert. Through thematic analysis, it became clear that incidents like phishing, password negligence, and unauthorized device use are not just operational failures but emotionally significant moments requiring judgment, adaptability, and emotional labor from supervisors.

The study’s clustering of supervisory responses into “unfavorable,” “challenge,” and “low-stress, low-effectiveness” categories—combined with multinomial logistic regression—identified psychological distress and time pressure as key predictors of suboptimal experiences. Conversely, perceived organizational cybersecurity support emerged as a protective factor, enabling supervisors to sustain effectiveness even under stress. These findings suggest that fostering a psychologically safe and procedurally supported work environment may be as critical to cyber resilience as technical controls.

The practical implications are far-reaching. The study emphasizes the need for aviation organizations to train supervisors not only in cybersecurity protocols but also in emotional regulation and critical incident response. Moreover, the identification of distinct psychological clusters and supervisory typologies underscores the value of predictive profiling and early-warning systems to detect supervisory overload before it compromises security or well-being. Finally, as aviation continues to digitalize, the study calls for a redefinition of cybersecurity leadership—one that recognizes supervisors as frontline agents of both technical enforcement and emotional stability. By presenting cybersecurity incidents as deeply human and psychologically disruptive events, this research contributes to both academic theory and sectoral practice. The aviation industry—and other safety-critical, digitally dependent sectors—must move beyond reactive models and embrace a proactive, integrated approach to cybersecurity leadership that centers on the people responsible for guiding teams through digital crises.

## Supporting information

S1 File Kopya cyber awareness survey data. (XLSX)
